# XDM-corrected hybrid DFT with numerical atomic orbitals predicts molecular crystal lattice energies with unprecedented accuracy†

**DOI:** 10.1039/d2sc05997e

**Published:** 2022-12-15

**Authors:** Alastair J. A. Price, Alberto Otero-de-la-Roza, Erin R. Johnson

**Affiliations:** a Department of Chemistry, Dalhousie University 6274 Coburg Rd Halifax B3H 4R2 Nova Scotia Canada erin.johnson@dal.ca; b Departamento de Química Física y Analítica and MALTA-Consolider Team, Facultad de Química, Universidad de Oviedo Oviedo 33006 Spain aoterodelaroza@gmail.com

## Abstract

Molecular crystals are important for many applications, including energetic materials, organic semiconductors, and the development and commercialization of pharmaceuticals. The exchange-hole dipole moment (XDM) dispersion model has shown good performance in the calculation of relative and absolute lattice energies of molecular crystals, although it has traditionally been applied in combination with plane-wave/pseudopotential approaches. This has limited XDM to use with semilocal functional approximations, which suffer from delocalization error and poor quality conformational energies, and to systems with a few hundreds of atoms at most due to unfavorable scaling. In this work, we combine XDM with numerical atomic orbitals, which enable the efficient use of XDM-corrected hybrid functionals for molecular crystals. We test the new XDM-corrected functionals for their ability to predict the lattice energies of molecular crystals for the X23 set and 13 ice phases, the latter being a particularly stringent test. A composite approach using a XDM-corrected, 25% hybrid functional based on B86bPBE achieves a mean absolute error of 0.48 kcal mol^−1^ per molecule for the X23 set and 0.19 kcal mol^−1^ for the total lattice energies of the ice phases, compared to recent diffusion Monte-Carlo data. These results make the new XDM-corrected hybrids not only far more computationally efficient than previous XDM implementations, but also the most accurate density-functional methods for molecular crystal lattice energies to date.

## Abstract 2

A new implementation of the XDM dispersion model within FHI-aims allows efficient use of XDM-corrected hybrid functionals, found to be the most accurate DFT methods for molecular crystal lattice energies to date.

## Graphical abstract

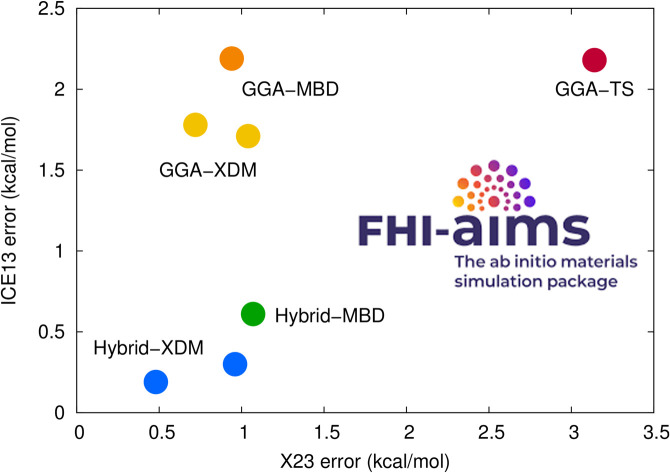

## 1 Introduction

The accurate description of molecular crystals is a challenge for current computational methods. Molecular crystal structures typically have unit cells containing hundreds of atoms, meaning a high computational expense, and feature a delicate balance between weak non-covalent (intermolecular) and strong covalent (intramolecular) interactions, both of which have to be described accurately by the chosen method. The computational description of these systems is important in the study of polymorphism, which is particularly prevalent in molecular crystals,^1,2^ pressure-temperature phase diagrams,^2^ and in any discipline in which the solid form of a molecular material controls a property of interest: pharmaceuticals (solubility/bioavailability and patentability^3–6^), foodstuffs (organoleptic properties^7^), energetic materials (sensitivity to detonation^8–10^), organic semiconductors (charge carrier mobility^11–15^), and others.^12^

Having a method that is able to rank molecular crystal structures accurately is essential for crystal structure prediction (CSP) – the prediction of the crystal structure of a compound from its molecular diagram only.^6,16,17^ A reliable CSP protocol would be extremely useful in the disciplines listed above, as it would allow circumventing experimental solid-form screening processes, which are expensive and time-consuming,^18–22^ and would provide a detailed energy–structure–function map for the chosen molecule and property of interest.^11,12^ To gauge progress in the field, the Cambridge Crystallographic Data Centre (CCDC) periodically runs CSP blind test competitions in which participant groups try to predict the observed crystal structures of a few molecular compounds.^23–28^ The 5th blind test, held in 2011, showed that final ranking of the candidate structures using dispersion-corrected DFT is quite effective, and far superior to force fields in most cases,^27,29–32^ and this conclusion was further supported by the 6th blind test.^28^ Although other techniques such as fragment-based methods,^2,33–37^ wavefunction theory,^38,39^ and machine-learning methods^37,40^ have been used, DFT is arguably the current workhorse for modeling molecular materials.^17,41–54^

Dispersion-corrected functionals based on the exchange-hole dipole moment (XDM) model,^55–58^ in particular the semilocal functional B86bPBE-XDM,^59,60^ have shown excellent performance for description of molecular crystals^44,45,57,61^ and non-covalent interactions in general.^62,63^ In its current plane-wave/pseudopotentials implementation, while still effective for CSP, B86bPBE-XDM is affected by outstanding drawbacks shared by all semilocal functionals. First, the use of a plane-wave basis set makes the computational requirements scale significantly with system size, such that calculations involving unit cells with hundreds to thousands of atoms are on the verge of being infeasible. Second, GGA functionals spuriously over-stabilize systems affected by delocalization error,^64–66^ which negatively impacts the modeling of molecular salts, acid–base co-crystals, hydrogen bonding, and halogen bonding, to list only a few examples.^44,67–69^ Lastly, GGA functionals give a poor description of conformational energies, which are important when comparing crystal polymorphs composed of flexible molecules.^45,70–72^ Notably, several studies have demonstrated the poor performance of B86bPBE-XDM for relative lattice energies in cases where delocalization error is prevalent,^44,45,70–72^ emphasizing the need for hybrid DFT.

In this work, we address these shortcomings by combining XDM functionals with the numerical atomic orbital (NAO) basis sets in the Fritz Haber Institute *ab initio* materials simulations (FHI-aims) package.^73–76^ FHI-aims offers near linear scaling with system size for self-consistent DFT calculations^73,74^ and enables relatively inexpensive use of hybrid functionals,^75^ compared to plane-wave approaches. This is important because hybrid functionals can be used to mitigate delocalization error^66,68,77–81^ and are generally more accurate than GGAs for conformational energies.^72^ One drawback of NAOs is the possible appearance of basis-set incompleteness error (BSIE), which is known to have a deleterious effect on the description of non-covalent interactions,^82–84^ although we show that BSIE can be effectively mitigated by parametrization of the dispersion damping function. Dispersion-corrected DFT methods with NAOs have been applied to molecular crystals in combination with the Tkachenko–Scheffler (TS)^85^ and many-body dispersion (MBD)^86,87^ family of corrections.^49–53^

To assess the new XDM-corrected hybrid functionals, we focus on molecular crystal lattice energies, as they are the key property for CSP ranking^88^ and one of the most demanding tests for computational methods regarding non-covalent interactions.^2^ The lattice energy of a molecular crystal is the energy required to separate the crystal at its equilibrium geometry into its component molecules. This is an essential parameter when assessing the accuracy of computational methods for modeling molecular crystals because the lattice energy is determined by a delicate balance between intermolecular and intramolecular interactions.^2^ The accurate calculation of lattice energies is also a stricter performance test for computational methods than energy differences between molecular crystal pairs because the benefits from error cancellation are minimized, while longer-range interactions and many-body effects become far more important. Here, we consider the lattice energies of the X23 set^35,38,39,57,89,90^ and of 13 ice phases, for which diffusion Monte Carlo (DMC) data has been generated.^91^ The latter is a particularly stringent test because determining accurate lattice energies for ice relies on a fine balance of dispersion, electrostatic, and many-body induction effects. At present, there is no functional that gives a good description of the absolute and relative energies of all ice phases,^91^ and therefore the reliable treatment of water and ice with DFT methods remains an unsolved problem.^91–93^

Herein, we show that the NAO implementation of XDM-corrected functionals provides excellent performance for the description of molecular dimers, ice, and molecular crystal lattice energies in general, with high computational efficiency. In particular, a composite method combining B86bPBE-XDM and its sequent 25% hybrid functional achieves mean absolute errors (MAEs) for the X23 and ice lattice energies of 0.48 kcal mol^−1^ and 0.19 kcal mol^−1^, respectively. For the X23, the reported MAE is roughly half the previous best value, making the new XDM methods the most accurate DFT approaches for modeling of molecular materials currently available.

## 2 Methods

### 2.1 Theory

A summary of the XDM dispersion model and its implementation in the FHIaims package is presented in this section. More details about the XDM method can be found in previous works (see ref. 58 and references therein). In XDM, the dispersion energy is calculated using a damped asymptotic pairwise dispersion expression,1
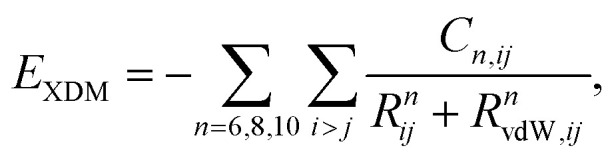
which is then added to the energy from the base density functional,

2 *E* = *E*_base_ + *E*_XDM_.

In eqn (1), *i* and *j* run over atoms, *R*_*ij*_ are the interatomic distances, *C*_*n*,*ij*_ are the dispersion coefficients, and the *R*_vdW,*ij*_ are damping lengths calculated as

3 *R*_vdW,*ij*_ = *a*_1_*R*_*c*,*ij*_ + *a*_2_,

with4
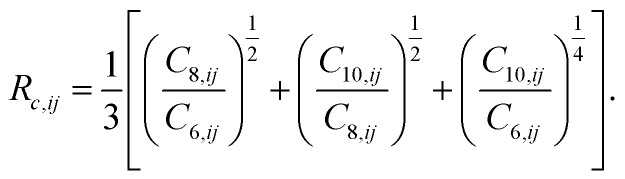


The *a*_1_ and *a*_2_ parameters are the damping function coefficients, which are determined for every functional and basis set combination by minimizing the root-mean-square percent error in binding energies for 49 small molecular dimers, relative to high-level reference data (the Kannemann–Becke set,^77,94,95^ KB49). The damping function is therefore used to match the XDM dispersion contribution to the particular exchange-repulsion behavior of the chosen functional, as well as to mitigate any (moderate) BSIE from an incomplete basis set. BSIE generally causes some overbinding of intermolecular dimers provided reasonable basis sets of at least double-ζ quality are used. Therefore, if the dispersion energy is damped slightly more strongly for an incomplete basis set, the overall binding energies can provide a good approximation to basis-set limit results. Importantly, once the *a*_1_ and *a*_2_ parameters are determined, they remain the same for every system to which the functional and basis set are applied, molecular or periodic, and are never re-parametrized for specific cases.

The dispersion coefficients in eqn (1) (*C*_*n*,*ij*_) are calculated non-empirically from the self-consistent electron density, its derivatives, and the kinetic energy density. It has been shown that the dependence of these coefficients on the chemical environment (the electronic many-body dispersion effects) is essential to the accuracy of the XDM method.^96^ Calculation of three-body and higher-order dispersion coefficients, of which the Axilrod–Teller–Muto (*C*_9_) is the leading term, is possible in XDM,^97^ but we have found that including this term has either little impact or degrades the accuracy of XDM-corrected functionals.^96^

The performance of an XDM-corrected method depends critically on the base functional with which it is paired. In this article, we consider two generalized-gradient-approximation (GGA) functionals: PBE,^60^ due to its popularity in the solid-state community, and B86bPBE,^59,60^ which is our GGA functional of choice when non-covalent interactions are dominant, thanks to its ability to accurately describe non-bonded repulsion.^57,62,95,98,99^ In addition, we consider multiple hybrid density functionals with exchange-correlation (XC) energies of the form

5 *E*_XC_ = (1 − *a*_X_)*E*^PBE/B86b^_X_ + *a*_X_*E*^SD^_X_ + *E*^PBE^_C_.

The exchange GGA is either PBE or B86b, *a*_X_ controls the fraction of exact exchange used in the functional, and the correlation contribution comes from PBE. We note that *E*^SD^_X_ is the exchange energy obtained using the exact formula for a single Slater determinant (as in Hartree–Fock theory) with the self-consistent orbitals as input. The PBE0 functional^100^ corresponds to the choice of PBE exchange and *a*_X_ = 0.25. Functionals with 50% exact exchange (“half-and-half”) have been shown to minimize delocalization error,^67,68,101^ so we also considered “PBE-50” with PBE exchange and *a*_X_ = 0.5. Given the good behavior of B86bPBE for intermolecular closed-shell repulsion, we define 25% and 50% hybrids built on B86b exchange as well, termed B86bPBE-25 and B86bPBE-50, respectively.^68^ Finally, we included the range-separated HSE06 hybrid functional^102^ as its use is fairly common in solid-state applications.

### 2.2 Computational details

All calculations in this work were carried out with the FHI-aims program (version 210513). The XDM method, B86b exchange, and the ensuing hybrid functionals, were all implemented in a copy of the code. The basis sets used for the calculations were either the “light” or the “tight” settings, which correspond to double-*ζ* and triple-*ζ* basis sets, respectively. Based on our initial exploration, the choice of integration mesh can substantially affect the stability of the geometry optimization procedure for molecular crystals. We therefore chose to always use the integration meshes from the tight settings, with up to 434 angular grid points.

The memory requirements of hybrid functional calculations with the tight basis set exceeded our current computational resources, so we approximated the hybrid/tight result using a correction calculated by evaluating the energy difference between tight and light bases at the GGA level:

6 *E*^tight^_hybrid_ ≈ *E*^light^_hybrid_ + (*E*^tight^_GGA_ − *E*^light^_GGA_).

This type of basis-set correction is analogous to using the difference between large- and small-basis MP2 energies to correct small-basis CCSD(T) energies, as in common practice in wavefunction theory calculations.^103^ In addition to the XDM-corrected functionals mentioned above, we also considered the Tkachenko–Scheffler (TS)^85^ and many-body dispersion (MBD)^86,87^ methods for comparison, since they are already implemented in FHIaims and are routinely used for molecular crystals and CSP.^49–53^ In the case of MBD, we used MBD@rsSCS^87^ as recommended by the FHI-aims documentation. In the rest of the article, MBD@rsSCS is referred to simply as MBD.

All calculations for the KB49,^77,94^ S22×5,^104^ and S66×8 ^105,106^ benchmarks of gas-phase dimer binding energies, as well as the 3B-69^107^ set of three-body interaction energies in molecular trimers, were carried out as single-point energy evaluations at the literature geometries. This is standard for these benchmark sets and is done to facilitate direct comparison with the CCSD(T) reference data. Since the S22×5, S66×8, and 3B-69 all contain dimer and trimer geometries far from equilibrium, geometry optimization would be meaningless for these systems. Conversely, full geometry optimizations were performed with each functional on both the molecular crystals and isolated molecules forming the X23 set^89,90,108^ of lattice energies. The geometries of the 13 ice polymorphs forming the ICE13 set^91^ were also fully optimized, although the geometry of the isolated water molecule was kept fixed, as described in ref. 91, for consistency. For the crystals, reciprocal-space *k*-point grids were selected with the number of points, *n*_1_ × *n*_2_ × *n*_3_, given by

7 *n*_*i*_ = int[max(1,*R*_*k*_|*b*_*i*_| + 0.5)],

where |*b*_*i*_| is the length of the *i*th reciprocal lattice vector and *R*_*k*_ = 50 bohr.

## 3 XDM parametrization

Before using the new FHIaims XDM implementation, we first need to parametrize the XDM damping function (eqn (3)) and find the optimal *a*_1_ and *a*_2_ for all chosen functional and basis set combinations. This is done in the same way as in previous studies, by minimizing the root-mean-square percent (RMSP) error in the binding energies of the 49 molecular dimers comprising the Kannemann–Becke set.^77,94^ The optimal parameter values, along with the resulting KB49 error statistics, are collected in Table 1. It is important to note that these *a*_1_ and *a*_2_ values are fixed for each particular functional and basis set combination, and do not change with the system to which XDM is applied.

**Table 1:** Optimal XDM parameters (*a*_1_ and *a*_2_) for selected functionals, with exact-exchange mixing fractions (*a*_X_) indicated^a^

Functional	*a*_X_	*a*_1_	*a*_2_ (Å)	MAE	MAPE
**Light basis set**					
PBE	0.00	0.5312	2.3270	0.67	19.0
B86bPBE	0.00	0.8219	1.2069	0.54	14.9
HSE06	0.11^b^	0.3268	3.0431	0.52	13.6
PBE0	0.25	0.3302	3.0042	0.46	12.5
PBE-50^c^	0.50	0.0000	4.1971	0.38	9.8
B86bPBE-25	0.25	0.5235	2.1995	0.35	9.7
B86bPBE-50	0.50	0.0831	3.7362	**0.30**	**8.5**
**Tight basis set**					
PBE	0.00	0.6438	1.8533	0.50	14.1
B86bPBE	0.00	0.8976	0.8518	0.38	11.0
HSE06	0.11^b^	0.5020	2.3000	0.46	11.1
PBE0	0.25	0.5053	2.2527	0.41	10.2
PBE-50	0.50	0.3983	2.5986	0.42	9.6
B86bPBE-25	0.25	0.6546	1.6097	**0.32**	**8.4**
B86bPBE-50	0.50	0.4887	2.1855	0.36	8.5

a The mean absolute errors (MAE, in kcal mol^−1^) and mean absolute percent errors (MAPE) for the KB49 fit set are also shown. The best overall results for each basis set are indicated in bold.

b This value is the range-separation parameter (*ω*) instead of the exact-exchange fraction.

c The optimal *a*_1_ value was negative, so it was set to zero during the parametrization.

The errors shown in Table 1 are comparable to those obtained with our previous plane-wave (Quantum ESPRESSO^57,109^) and Gaussian basis-set (using Gaussian^110^ or psi4 ^111^ with the postg program^112^) results contained in the current XDM parametrization database.^113^ For example, the MAPE for B86bPBE/tight in Table 1 (11.0%) is very close to the MAPE obtained for the same functional using the projector augmented wave (PAW) method^114^ (11.8%), plane waves plus norm-conserving pseudopotentials (12.4%), and the aug-cc-pVTZ Gaussian basis set (11.4%). The MAPEs obtained with other functionals, such as PBE, PBE0, or HSE06, also deviate from those in the parametrization database by around 1% at most. This is a strong indication that our FHIaims XDM implementation is working correctly.

Focusing on the results for the tight basis set, Table 1 shows that hybrid functionals outperform GGAs, and that B86b-based functionals consistently give lower errors than the analogous PBE-based functionals. This is also in agreement with our previous works.^57,77^ The lowest errors among the functionals studied are obtained for the B86bPBE-25 hybrid, with a MAE of 0.32 kcal mol^−1^ and a MAPE of 8.4%.

Because the tight basis set is too expensive for routine geometry optimizations, we resort to using the smaller, light basis set and relying on the XDM damping function to partially alleviate any BSIE.^77,82^Table 1 also shows the average errors for the light (double-*ζ*) basis set. While lower errors are obtained with the tight (triple-*ζ*) basis set, the good performance of the light basis set indicates a reasonably low impact on the accuracy caused by BSIE. This is in stark contrast to our previous results using the double-*ζ* basis set in the SIESTA NAO code,^115,116^ where the MAPE was in the 20% to 30% range and could not mitigated by using counterpoise corrections. The differences between FHI-aims and SIESTA are likely due to the different strategies employed by their developers for NAO construction (see ref. 73 and 115 for details). The small magnitude of the BSIE with FHI-aims can be confirmed by comparing the dispersion-uncorrected binding energies calculated with the light and tight basis sets using the same functional. For example, the mean absolute difference between the light and tight binding energies obtained using B86bPBE (without XDM) is 0.32 kcal mol^−1^, with individual errors not exceeding 0.75 kcal mol^−1^.

## 4 Molecular benchmarks

In order to build a method that works reliably for molecular crystals, it is imperative to avoid error cancellation as much as possible. Therefore, it is interesting to examine whether individual interactions between monomer pairs are accurately represented. For this reason, we first evaluate the performance of the new implementation of XDM for selected molecular benchmarks comprising gas-phase dimers, and compare it to the TS^85^ and MBD^86,87^ dispersion corrections also implemented in FHI-aims. We consider the S22×5 ^104^ and S66× 8^105,106^ benchmarks, which comprise non-covalent interaction energies of small molecular dimers at and around their equilibrium geometries. It is worth noting that the single damping parameter employed in MBD was fit to minimize the mean absolute relative error in the S66×8 binding energies,^87^ although this parameter was only fit for use with the tight setting (termed the “tier 2” basis set in ref. 87) and, unlike the XDM damping parameters, is not basis-set dependent. TS and MBD are paired only with the PBE, HSE06, and PBE0 functionals for which damping parameters are available.

The S22×5 and S66×8 error statistics for the various combinations of functional, basis set, and dispersion correction are shown in Table 2. As for the KB49 set, the average errors are lower for the tight basis set, and hybrid functionals slightly outperform GGAs regardless of the dispersion correction employed. The XDM values in the table are also similar to those reported for the same benchmarks using the aug-cc-pVTZ Gaussian basis set.^58^ While all basis set, functional, and dispersion method combinations perform generally well, B86bPBE-25-XDM consistently gives the lowest errors by a small margin, with MAEs in the range of 0.2–0.4 kcal mol^−1^.

**Table 2:** Mean absolute errors (in kcal mol^−1^) for the S22×5, ^104^ S66×8,^105^ and 3B-69 ^107^ molecular benchmarks using selected functionals and dispersion corrections^a^

Functional	Dispersion	S22×5		S66×8		3B-69	
	Correction	Light	Tight	Light	Tight	Light	Tight
PBE	TS	0.57	0.39	0.60	0.38	0.078	0.080
HSE06	TS	0.63	0.45	0.64	0.38	0.046	0.042
PBE0	TS	0.58	0.42	0.59	0.33	0.044	0.039
PBE	MBD	0.55	0.44	0.44	0.28	0.113	0.113
HSE06	MBD	0.53	0.48	0.45	0.29	0.069	0.066
PBE0	MBD	0.50	0.46	0.40	0.26	0.060	0.055
PBE	XDM	0.58	0.44	0.45	0.29	0.101	0.099
B86bPBE	XDM	0.46	0.34	0.35	0.20	0.050	0.052
HSE06	XDM	0.52	0.45	0.41	0.28	0.054	0.055
PBE0	XDM	0.49	0.43	0.38	0.25	0.044	0.045
PBE-50	XDM	0.47	0.47	0.37	0.28	0.047	**0.030**
B86bPBE-25	XDM	**0.39**	**0.35**	**0.30**	**0.19**	**0.037**	0.040
B86bPBE-50	XDM	0.40	0.41	0.32	0.24	0.055	0.051

a The best overall results in each column are indicated in bold.

Beyond-pairwise intermolecular interactions are also important in molecular crystals, since they represent a small but significant fraction of the total lattice energy.^2,117^ For this reason, we consider as an additional benchmark the 3B-69 set of molecular trimers.^107^ In this case, the reference data corresponds to the difference between the trimer binding energy and the pairwise sum of the constituent dimer binding energies. This is a good measure of whether the considered methods can describe non-additive many-body intermolecular interactions^96^ and, as such, highlight their performance in the treatment of beyond-pairwise effects. Table 2 shows that BSIE has less impact on the three-body energies than it does for the pairwise binding energies, with the light and tight MAEs being approximately the same.

MBD might be expected to be the most accurate dispersion correction for the 3B-69 benchmark due to the many-body nature of the interactions. However, we observe that all three dispersion methods provide roughly comparable performance, with XDM being slightly superior to MBD for the same functional and basis set combination. Instead, it is the choice of base functional that is the determining factor, with PBE consistently giving the largest errors, while use of either B86b or exact exchange improves performance in the treatment of three-body interactions. This confirms our previous observation that the choice of base functional is critical for accurate treatment of beyond-pairwise non-covalent interactions.^118^ Overall, XDM paired with either the B86bPBE-25 or PBE-50 hybrid functionals (depending on basis set) gives the lowest MAE. The fact that XDM (which does not incorporate a three-body dispersion contribution) outperforms MBD (which does) for the description of three-body intermolecular interactions suggests that electronic many-body effects are much more important than the atomic many-body dispersion effects encapsulated by the Axilrod–Teller–Muto term.^96^

## 5 X23 lattice energies

Reference lattice energies for molecular crystals are typically derived from experimental sublimation enthalpies^119^ using a back-correction for vibrational effects.^35,38,39,57,89,90^ The X23 set,^89,108^ which comprises 23 reference lattice energies, has become the standard benchmark and DFT methods have been extensively tested using this set.^38,57,89,120,121^ Here, we use the most recent re-determination of the X23 reference data^90^ to assess the performance of the various functionals and dispersion corrections examined in this work. The error statistics are shown in Table 3.

**Table 3:** Mean absolute errors (in kcal mol^−1^) for the X23 solid-state benchmark with selected functionals, dispersion corrections, and basis sets

Functional	Dispersion	Light	Tight
**Full geometry relaxation**			
PBE	TS	4.17	3.14
HSE06	TS	4.57	—
PBE0	TS	4.44	2.39^a^
PBE	MBD	1.61	0.94
HSE06	MBD	2.12	—
PBE0	MBD	1.98	0.84^a^
PBE	XDM	1.14	1.04
HSE06	XDM	1.20	—
PBE0	XDM	1.14	—
PBE50	XDM	1.25	—
B86bPBE	XDM	0.83	**0.72**
B86bPBE-25	XDM	**0.81**	—
B86bPBE-50	XDM	1.06	—
**Single points at GGA/light geometries**			
PBE0//PBE	MBD	1.97	1.07^b^
PBE0//PBE	XDM	1.01	0.96^b^
PBE-50//PBE	XDM	1.00	0.87^b^
B86bPBE-25//B86bPBE	XDM	**0.66**	**0.48** b
B86bPBE-50//B86bPBE	XDM	0.70	0.53^b^

a Literature value obtained from ref. 87.

b The hybrid energies with the light settings are corrected using the difference between light and tight results at the GGA level (*via*eqn (6)).

The table is separated into two sections, with the upper part showing results obtained with full geometry optimization of the molecular crystals at each listed level of theory. As noted in the computational methods section, we were only able to perform calculations using the tight basis set for GGA functionals due to the high memory requirements for hybrids. However, literature results^87^ for PBE0-TS and PBE0-MBD with tight settings (which used the earlier X23 reference data^89^) are provided as these combinations give the lowest MAEs obtained with each of these dispersion corrections. We note that updating the reference data causes the MAEs to change by at most 0.25 kcal mol^−1^, although often the deviation is lower.

As observed previously,^87,108^ TS massively overbinds these molecular crystals. With TS and MBD, there is a significant difference between the light and tight results, which occurs because the damping parameters within these dispersion corrections are not optimized for each basis set independently. As a result, XDM significantly outperforms MBD with the light basis set, although the two methods give comparable MAEs with the tight basis. Also, the PBE-XDM and B86bPBE-XDM MAEs with light are in excellent agreement with previous results obtained using the Quantum ESPRESSO plane-wave code.^62^

B86bPBE-XDM with the tight basis set yields the lowest MAE (0.72 kcal mol^−1^) yet obtained for the X23 set with any dispersion-corrected GGA, although this is largely due to the improvement in the reference data (the MAE compared to the values in ref. 89 is 0.90 kcal mol^−1^). For comparison, the MAE for PBE0-MBD in Table 3 is 0.84 kcal mol^−1^ and the lowest MAE reported by Thomas *et al.*^122^ for the X23 is 0.81 kcal mol^−1^, obtained with the TPSS-D3 dispersion-corrected meta-GGA. (The D3 dispersion correction by Grimme *et al.* is not available in FHIaims, so a direct comparison was not possible.) The best GGA results given by Thomas *et al.*^122^ are 0.93 kcal mol^−1^ (B86bPBE-XDM with plane-waves, a slightly outdated XDM implementation, and only for the C21) followed by PBE-D3 at 0.98 kcal mol^−1^. Regarding the dispersion-corrected hybrid functionals, the best results are obtained with B86bPBE-25/light (0.81 kcal mol^−1^ with either the Dolgonos *et al.*^90^ or the older Reilly *et al.*^89^ reference data) followed by PBE0-MBD/tight (0.84 kcal mol^−1^), with the former being considerably more efficient. For comparison, Thomas *et al.*^122^ report MAEs of 0.93 kcal mol^−1^ for PBE0-MBD at the PBE-TS optimized geometries and 1.03 kcal mol^−1^ for PBE0-D3.

Computational efficiency is an important consideration in CSP, where hundreds to thousands of candidate crystal structures must be ranked with DFT for a given compound. Composite approaches, in which a relatively low level of theory is used for geometry optimization, followed by single-point energy evaluation at a higher level of theory, are an excellent strategy to reduce the computational cost without losing accuracy.^46,123^ In this work, we consider composite approaches that use dispersion-corrected GGA functionals (PBE-MBD, PBE-XDM, or B86bPBE-XDM) and the light basis set for geometry optimization. Single-point energies are then evaluated with the corresponding 25% or 50% hybrid functionals and the light basis set and, in some cases, also with the same GGA and the tight basis set. This allows us to obtain energies (*via*eqn (6)) with an accuracy comparable to what would be expected from full hybrid/tight calculations, but with a drastically reduced computational cost. MAEs in the X23 lattice energies obtained using this type of composite approach are shown in the lower portion of Table 3. The notation in the table is high-level (hybrid)//low-level (GGA).

The MAEs obtained with the composite approach using B86bPBE-25-XDM and B86bPBE-50-XDM are the lowest errors yet obtained for the X23 set with any DFT method. The composite B86bPBE-25-XDM//B86bPBE-XDM approach with basis-set correction gives an MAE of only 0.48 kcal mol^−1^, well below the usual target of 1 kcal mol^−1^ deemed to be chemical accuracy and almost exactly on the 2 kJ mol^−1^ mark commonly cited as the average energy difference between polymorphs.^124^ It is also reassuring that the average error for molecular crystals is similar to that for dimers formed from molecules with similar sizes (Table 2), for which the MAEs were in the 0.2 kcal mol^−1^ to 0.4 kcal mol^−1^ range.

**Fig. 1:**
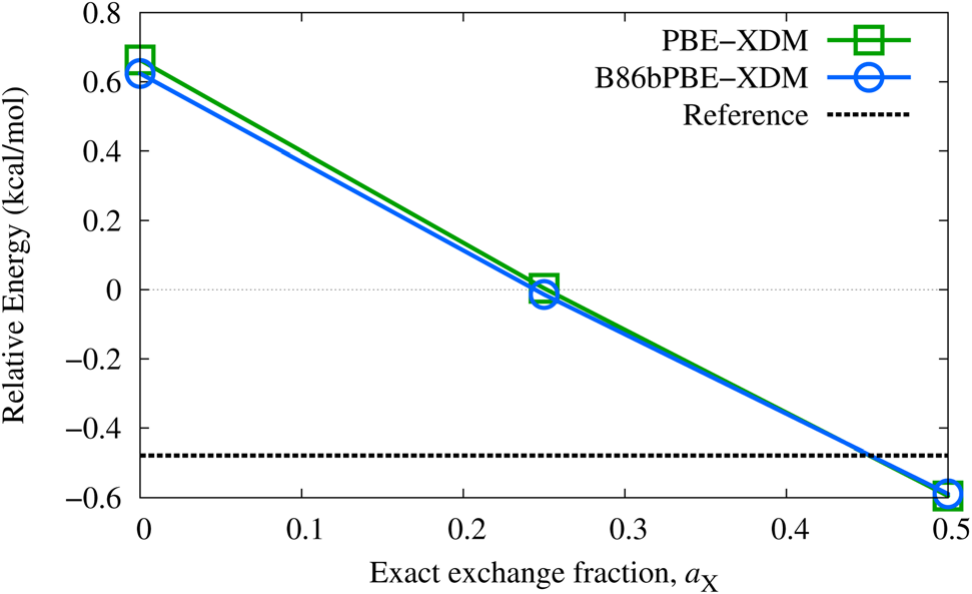
Relative energies of the α and β polymorphs of oxalic acid computed with various XDM-corrected GGA and hybrid functionals with the light basis set, evaluated at the corresponding GGA geometries. The α form is the most stable experimentally.

While good performance for absolute lattice energies is highly desirable, it does not necessarily ensure reliable polymorph ranking, which is dependent on accurate lattice-energy differences (as well as thermal and kinetic factors). The performance of the proposed methods for relative lattice energies will be examined in detail elsewhere. Nonetheless, improvements in absolute lattice energies do tend to result in more accurate relative lattice energies, as seen for the two oxalic acid polymorphs (α and β forms) appearing in the X23 set. The choice of dispersion correction has only a minor effect on this energy difference, although the polymorph ordering is highly dependent on exact-exchange mixing in the base functional. As shown in Fig. 1, the GGAs predict the incorrect energy ordering and relatively large fractions of exact exchange (near 50%) are needed to recover the reference lattice-energy difference. This suggests that delocalization error is a factor in determining the most stable oxalic acid polymorph, with the β form likely favored by GGAs due to its dimeric hydrogen-bonding.

## 6 Ice lattice energies

Lastly, we examine the calculation of the lattice energies for the various phases of ice. The study of intermolecular interactions in water is both very important, because of its central role in many disciplines, and very challenging computationally, as electrostatics, induction, and dispersion all play a role. In general, it is agreed that the dispersion contribution, albeit smaller than in other non-covalently bound systems, is still necessary to describe water-water interactions accurately.^91,92^ There are also significant many-body effects in water arising from intermolecular electron delocalization^93^ that lead to delocalization error. As a result, a functional that describes the properties of water and ice accurately is still missing.^91,93^

A strict test of density functionals and dispersion corrections for water is calculation of the absolute lattice energies of the various (ordered) ice phases. Different ice phases vary in molecular arrangements and in the extent of electron delocalization, which has been shown to correlate with the absolute lattice energy.^125^ In a recent work, Della Pia *et al.*^91^ reported absolute lattice energies of 13 ordered ice phases calculated using Diffusion Monte Carlo (DMC), and subsequently benchmarked a number of functionals using a plane-wave approach. Relative to the X23, this set has the advantage that no vibrational or nuclear quantum effects need to be removed before comparison to DFT results. We now use this ICE13 set, which is a superset of the previous ICE10 set proposed by Brandenburg *et al.*,^92^ to evaluate the performance of our XDM-corrected methods.

Table 4 shows the MAEs calculated for the absolute and relative lattice energies of the ICE13 set with respect to the DMC data. The MAE of the relative energies is calculated by considering all 78 pairs of crystals in the ICE13 set, to avoid singling out any particular ice phase. As in the case of the X23, the MAEs with the tight basis set are lower (in most cases) than with light, both for the absolute and for the relative lattice energies. XDM outperforms TS and MBD for absolute lattice energies by around 0.4–0.5 kcal mol^−1^, but gives higher errors by a few tenths of a kcal mol^−1^ for the relative lattice energies. The average errors from the GGA functionals are quite high, in the vicinity of 2 kcal mol^−1^ for the absolute lattice energies and *ca.* 0.5 kcal mol^−1^ or more for the relative lattice energies. Hybrids give improved results, providing another indication that the cooperative hydrogen bonding networks in ice exhibit considerable delocalization error.

**Table 4:** Mean absolute errors (in kcal mol^−1^) for the ICE13 ice phases benchmark with selected functionals, dispersion corrections, and basis sets

Functional	Dispersion	Absolute		Relative	
	Correction	Light	Tight	Light	Tight
**Full geometry relaxation**					
PBE	TS	3.69	2.18	0.56	0.51
HSE06	TS	2.68	—	0.40	—
PBE0	TS	2.38	—	**0.37**	—
PBE	MBD	3.70	2.19	0.66	0.60
HSE06	MBD	2.68	—	0.40	—
PBE0	MBD	2.38	—	**0.37**	—
PBE	XDM	2.79	**1.71**	0.91	0.73
HSE06	XDM	1.60	—	0.72	—
PBE0	XDM	1.35	—	0.65	—
PBE50	XDM	0.79	—	0.46	—
B86bPBE	XDM	2.69	1.78	0.67	**0.45**
B86bPBE-25	XDM	1.16	—	0.53	—
B86bPBE-50	XDM	**0.55**	—	0.42	—
**Single points at GGA/light geometries**					
PBE0//PBE	MBD	2.13	0.61^a^	0.34	0.29^a^
PBE0//PBE	XDM	1.11	0.30^a^	0.61	0.43^a^
PBE-50//PBE	XDM	**0.25**	1.16^a^	0.35	0.21^a^
B86bPBE-25//B86bPBE	XDM	0.93	**0.19** a	0.49	0.28^a^
B86bPBE-50//B86bPBE	XDM	0.32	1.20^a^	0.31	**0.19** a

a The hybrid energies with the light settings are corrected using the difference between light and tight results at the GGA level (*via*eqn (6)).

While we could not run the hybrid calculations with the tight basis set, the light results indicate that 25% hybrid functionals reduce the MAE, and 50% hybrids reduce it even further. However, the statistics for the composite methods in Table 4 allow us to understand the effects of BSIE and the incorporation of exact exchange separately, and reveal that this may be ascribed to error cancellation. While the results with the 25% hybrids improve when the basis-set correction of eqn (6) is added, the 50% hybrid functionals perform better with the light basis set and no BSIE correction. This suggests that, when half-and-half functionals are used for water, there is error cancellation between delocalization error and BSIE.

As for the X23, the best-performing method for ice is found to be the composite approach using B86bPBE-25-XDM with the additive BSIE correction, which yields MAEs of 0.19 kcal mol^−1^ and 0.28 kcal mol^−1^ for the absolute and relative lattice energies, respectively. This absolute lattice-energy error is lower than the MAEs of all functionals studied by Della Pia *et al.*,^91^ and the relative-energy error is also among the best. For comparison, the best-performing functional reported^91^ for absolute lattice energies is the revPBE-D3 GGA, with a MAE of 0.22 kcal mol^−1^, and the best functionals in each of the other classes are rSCAN (meta-GGA, 0.23 kcal mol^−1^), vdw-DF2 (non-local, 0.32 kcal mol^−1^), and revPBE0-D3 (hybrid, 0.39 kcal mol^−1^). Naturally, all these functionals are well within the “good functional” category established by the authors (MAEs <0.96 kcal mol^−1^ and <0.48 kcal mol^−1^ for absolute and relative lattice energies, respectively).

## 7 Conclusions

The calculation of lattice energies, the energy required to separate a molecular crystal into its component molecules, is of fundamental importance and a particularly stringent test for computational methods. The plane-wave implementation of exchange-hole dipole moment (XDM) dispersion model, in particular in combination with the B86bPBE functional, has been shown to give excellent results for the calculation of absolute and relative lattice energies. This makes it a good choice for the final energy ranking in molecular crystal structure prediction (CSP). However, the reliance on plane waves imposes a poor computational scaling with system size and limits the applicability of XDM to semilocal functionals, which results in poor performance for systems with high conformational flexibility or significant delocalization error. In this work, we presented the implementation of XDM with numerical atomic orbitals (NAO) in the FHIaims package. This enables the efficient combination of XDM with hybrid functionals without significant basis-set incompleteness errors, thus mitigating the aforementioned problems.

To test the accuracy of the new XDM-corrected hybrid functionals, we assessed their performance for binding energies of molecular gas-phase dimers and trimers, as well as lattice energies of small molecular crystals (the X23 set) and 13 phases of ice (the ICE13 set). The results were compared to the Tkachenko–Scheffler (TS) and state-of-the-art many-body dispersion (MBD@rsSCS) methods. For molecular dimers, XDM-corrected functionals achieve a mean average error (MAE) of between 0.2 and 0.4 kcal mol^−1^, slightly outperforming TS and MBD. More importantly, XDM-corrected functionals also show excellent performance for three-body interaction energies (the 3B-69 set), suggesting that electronic many-body effects are much more important than atomic many-body dispersion effects, which are not included in the canonical XDM methods.

The XDM-corrected methods also yield very low average errors for the X23 set of lattice energies, particularly if hybrid methods or relatively large (“tight”) basis sets are used. The most intriguing result is the spectacular performance of composite methods, in which a GGA geometry optimization (*e.g.* B86bPBE-XDM) is followed by a single-point energy calculation to incorporate the benefits of using a hybrid functional (*e.g.* B86bPBE-25-XDM), and perhaps an additional single-point correction to treat basis-set incompleteness error (the difference between tight and light energies at the GGA level). The best-performing composite method (B86bPBE-25-XDM with basis-set correction, at the B86bPBE-XDM equilibrium geometries) achieves a MAE of only 0.48 kcal mol^−1^ for the X23 set, roughly half the error of other similar DFT methods. Moreover, this composite approach can be routinely applied to molecular crystals containing as many as 1000 atoms within the unit cell.

The excellent performance of the basis-corrected B86bPBE-25-XDM//B86bPBE-XDM composite method extends to the calculation of the absolute lattice energies of 13 ice phases, for which it achieves an MAE of only 0.19 kcal mol^−1^, outperforming all DFT functionals reported to date. The calculation of absolute lattice energies of ice is particularly difficult due to the presence of delocalization error and the delicate balance between electrostatics, dispersion, and induction. It is an key point that one single methodology works well for molecular dimers and trimers, and achieves the lowest MAE for both the X23 and ICE13 lattice energies. Obtaining good across-the-board performance in all tests examined is of paramount importance when modeling complex materials that feature several disparate types of non-covalent interactions. This makes us confident that the proposed XDM-corrected methods will serve nicely for accurate energy ranking in crystal structure prediction.

## Data availability

The data that support the findings of this study are available from the corresponding author upon reasonable request.

## Author contributions

Coding was done by AJAP with assistance from AOR and ERJ. AJAP also performed the calculations. All three authors shared equally in data analysis and writing of the manuscript.

## Conflicts of interest

There are no conflicts to declare.

## Supplementary Material

SC-014-D2SC05997E-s001

SC-014-D2SC05997E-s002

SC-014-D2SC05997E-s003
