# Supplementary material for: XDM-corrected hybrid DFT with numerical atomic orbitals predicts molecular crystal lattice energies with unprecedented accuracy
Source: Chem Sci. 2022 Dec 15;14(5):1252–62. doi: 10.1039/d2sc05997e (PMC9891363; doi:10.1039/d2sc05997e)
Supplement: SC-014-D2SC05997E-s003 [file SC-014-D2SC05997E-s003.pdf]

Electronic Supplementary Material (ESI) for Chemical Science.
This journal is © The Royal Society of Chemistry 2022

Supplementary Information for: XDM-corrected hybrid DFT with numerical atomic orbitals predicts molecular crystal lattice energies with unprecedented accuracy

Alastair J. A. Price, Alberto Otero de la Roza, and Erin R. Johnson

November 24, 2022

Table S1: Effect of using tight, as opposed to light, geometries for composite calculations for the X23 and ICE13 benchmarks. Results shown are mean absolute errors in the computed lattice energies, in kcal/mol.

Method	X23		ICE13	
	tight@light	tight@tight	tight@light	tight@tight
PBE0//PBE (MBD)	1.07	1.03	0.61	0.71
PBE0//PBE (XDM)	0.96	0.94	0.30	0.33
PBE-50//PBE (XDM)	0.87	0.86	1.16	0.98
B86bPBE-25//B86bPBE (XDM)	0.48	0.50	0.19	0.22
B86bPBE-50//B86bPBE (XDM)	0.53	0.54	1.20	1.02

Table S2: X23 and ICE13 lattice energies, in kcal/mol, computed with selected methods using fully relaxed geometries.

Name	PBE-TS light	PBE-TS tight	HSE06-TS light	PBE0-TS light
1,4-cyclohexanedione	26.382	25.460	27.081	27.031
acetic acid	20.391	19.687	20.622	20.454
adamantane	26.387	26.299	26.697	26.766
ammonia	11.340	10.673	10.818	10.622
anthracene	34.536	32.506	36.219	36.261
benzene	16.871	16.042	17.233	17.261
CO_2	6.960	6.105	7.096	7.008
cyanamide	22.896	22.555	22.284	22.041
cytosine	43.105	41.426	44.001	43.735
ethylcarbamate	24.607	23.838	24.791	24.595
formamide	21.517	20.609	21.579	21.357
hexamine	28.913	27.590	29.335	29.342
imidazole	25.557	24.404	25.452	25.361
naphthalene	25.564	24.167	26.612	26.641
α oxalic acid	25.696	23.970	27.008	26.684
β oxalic acid	26.455	24.738	27.040	26.732
pyrazine	19.474	18.075	19.731	19.636
pyrazole	22.480	21.263	22.460	22.352
succinic acid	36.958	35.553	37.171	36.951
triazine	17.500	16.429	17.943	17.945
trioxane	19.356	18.163	19.719	19.661
uracil	37.092	35.590	38.062	37.785
urea	27.354	26.824	27.683	27.345
MAE	4.171	3.140	4.568	4.436
ME	4.166	3.060	4.568	4.434
ice Ih	18.248	16.675	17.003	16.650
ice II	17.790	16.292	16.812	16.509
ice III	17.895	16.324	16.762	16.432
ice IV	17.311	15.811	16.352	16.066
ice VI	17.290	15.814	16.357	16.081
ice VII	15.525	14.112	14.831	14.619
ice VIII	15.833	14.451	15.129	14.921
ice IX	18.018	16.478	16.918	16.587
ice XI	18.322	16.746	17.071	16.717
ice XIII	17.733	16.233	16.728	16.426
ice XIV	17.613	16.112	16.620	16.327
ice XV	17.290	15.814	16.357	16.081
ice XVII	17.916	16.369	16.702	16.354
MAE	3.686	2.181	2.675	2.377
ME	3.686	2.181	2.675	2.377

Table S3: X23 and ICE13 lattice energies, in kcal/mol, computed with selected methods using fully relaxed geometries.

Name	PBE-MBD light	PBE-MBD tight	HSE06-MBD light	PBE0-MBD light
1,4-cyclohexanedione	22.918	22.092	23.829	23.755
acetic acid	19.070	18.397	19.277	19.106
adamantane	19.065	19.220	19.528	19.573
ammonia	10.977	10.284	10.428	10.221
anthracene	27.533	25.671	29.708	29.737
benzene	13.842	13.084	14.498	14.503
CO_2	6.514	5.636	6.769	6.694
cyanamide	22.564	22.225	21.911	21.660
cytosine	40.337	38.730	41.265	40.986
ethylcarbamate	22.613	21.936	22.907	22.690
formamide	20.680	19.779	20.752	20.520
hexamine	22.941	21.716	23.847	23.820
imidazole	23.551	22.423	23.572	23.468
naphthalene	20.492	19.221	21.925	21.945
α oxalic acid	24.670	22.971	26.049	25.714
β oxalic acid	25.243	23.549	26.017	25.702
pyrazine	16.360	15.017	16.757	16.651
pyrazole	20.933	19.735	21.057	20.943
succinic acid	34.256	32.926	34.677	34.446
triazine	14.720	13.715	15.307	15.301
trioxane	16.435	15.305	17.010	16.955
uracil	34.493	33.054	35.480	35.191
urea	26.854	26.323	27.270	26.916
MAE	1.608	0.936	2.121	1.982
ME	1.543	0.497	2.098	1.953
ice Ih	18.497	16.920	17.193	16.838
ice II	17.736	16.233	16.744	16.437
ice III	17.897	16.328	16.706	16.373
ice IV	17.235	15.730	16.298	16.003
ice VI	17.100	15.616	16.189	15.907
ice VII	15.683	14.250	15.082	14.840
ice VIII	15.937	14.535	15.358	15.114
ice IX	18.031	16.486	16.870	16.536
ice XI	18.568	16.988	17.257	16.902
ice XIII	17.584	16.081	16.565	16.261
ice XIV	17.420	15.914	16.423	16.128
ice XV	17.100	15.616	16.189	15.907
ice XVII	18.171	16.620	16.902	16.552
MAE	3.699	2.188	2.685	2.379
ME	3.699	2.188	2.685	2.379

Table S4: X23 and ICE13 lattice energies, in kcal/mol, computed with selected methods using fully relaxed geometries.

Name	PBE-XDM light	PBE-XDM tight	HSE06-XDM light	PBE0-XDM light	PBE50-XDM light
1,4-cyclohexanedione	19.743	20.397	19.653	19.816	20.101
acetic acid	17.274	17.414	16.983	16.915	16.780
adamantane	14.708	16.366	13.944	14.231	13.414
ammonia	10.030	9.782	9.238	9.113	8.445
anthracene	22.711	23.127	23.227	23.671	24.049
benzene	11.554	11.917	11.468	11.666	11.550
CO_2	6.228	5.830	6.183	6.172	6.113
cyanamide	21.025	21.378	20.088	19.962	19.237
cytosine	36.787	36.695	36.728	36.700	36.777
ethylcarbamate	19.978	20.352	19.537	19.480	19.119
formamide	19.247	19.047	18.893	18.764	18.477
hexamine	19.410	20.109	18.918	19.140	18.776
imidazole	21.628	21.414	21.076	21.106	20.622
naphthalene	16.893	17.333	17.149	17.475	17.630
α oxalic acid	22.459	21.863	23.074	22.927	23.807
β oxalic acid	23.122	22.522	23.147	22.995	23.434
pyrazine	14.621	14.398	14.303	14.410	14.141
pyrazole	18.908	18.648	18.441	18.475	18.098
succinic acid	30.707	30.930	30.067	30.032	29.911
triazine	13.350	13.375	13.271	13.444	13.536
trioxane	14.092	14.172	13.788	13.896	13.788
uracil	31.587	31.500	31.655	31.599	31.706
urea	24.549	24.909	24.481	24.280	24.241
MAE	1.135	1.043	1.203	1.143	1.253
ME	-0.912	-0.787	-1.142	-1.101	-1.210
ice Ih	17.898	16.605	16.500	16.181	15.419
ice II	16.794	15.717	15.623	15.375	14.822
ice III	17.140	15.935	15.801	15.512	14.802
ice IV	16.247	15.213	15.098	14.872	14.372
ice VI	16.055	15.065	14.930	14.716	14.224
ice VII	14.462	13.628	13.574	13.428	13.235
ice VIII	14.707	13.905	13.839	13.690	13.499
ice IX	17.240	16.056	15.925	15.635	14.927
ice XI	17.969	16.674	16.563	16.244	15.473
ice XIII	16.627	15.569	15.419	15.175	14.589
ice XIV	16.418	15.382	15.217	14.987	14.423
ice XV	16.054	15.065	14.929	14.716	14.223
ice XVII	17.578	16.306	16.217	15.903	15.174
MAE	2.794	1.711	1.597	1.351	0.793
ME	2.794	1.711	1.597	1.351	0.793

Table S5: X23 and ICE13 lattice energies, in kcal/mol, computed with selected methods using fully relaxed geometries.

Name	B86bPBE-XDM light	B86bPBE-XDM tight	B86bPBE25-XDM light	B86bPBE50-XDM light
1,4-cyclohexanedione	20.329	21.544	20.252	20.358
acetic acid	17.285	17.602	16.915	16.754
adamantane	15.612	17.897	14.901	13.863
ammonia	10.003	9.936	9.022	8.309
anthracene	24.266	25.252	24.994	24.985
benzene	12.074	12.779	12.115	11.871
CO_2	6.095	5.712	6.086	6.024
cyanamide	20.916	21.413	19.953	19.253
cytosine	37.400	37.771	37.226	37.142
ethylcarbamate	20.155	20.819	19.582	19.151
formamide	19.286	19.294	18.768	18.440
hexamine	20.907	22.703	19.947	19.096
imidazole	21.794	21.902	21.273	20.760
naphthalene	17.952	18.846	18.391	18.289
α oxalic acid	22.794	22.440	23.161	23.868
β oxalic acid	23.418	23.066	23.198	23.468
pyrazine	15.086	15.131	14.825	14.472
pyrazole	19.130	19.156	18.666	18.237
succinic acid	31.205	31.953	30.350	30.022
triazine	13.722	13.950	13.802	13.846
trioxane	14.808	15.272	14.282	13.930
uracil	31.997	32.202	31.972	31.986
urea	24.683	25.336	24.288	24.166
MAE	0.825	0.715	0.812	1.064
ME	-0.464	0.017	-0.766	-1.013
ice Ih	17.529	16.360	15.835	15.128
ice II	16.687	15.768	15.186	14.584
ice III	16.884	15.812	15.244	14.548
ice IV	16.216	15.364	14.723	14.146
ice VI	16.062	15.252	14.592	14.008
ice VII	14.707	14.140	13.417	13.041
ice VIII	14.957	14.418	13.679	13.304
ice IX	16.991	15.946	15.373	14.677
ice XI	17.600	16.429	15.897	15.182
ice XIII	16.533	15.631	14.998	14.362
ice XIV	16.371	15.499	14.835	14.202
ice XV	16.062	15.252	14.592	14.008
ice XVII	17.232	16.083	15.570	14.887
MAE	2.689	1.776	1.159	0.554
ME	2.689	1.776	1.159	0.554

Table S6: X23 and ICE13 lattice energies, in kcal/mol, computed with selected methods using GGA/light (PBE-MBD or PBE-XDM) geometries.

Name	PBE-MBD tight	PBE0-MBD light	PBE-XDM tight	PBE0-XDM light	PBE50-XDM light
1,4-cyclohexanedione	22.093	23.778	20.364	19.986	20.506
acetic acid	18.408	19.111	17.413	16.991	16.975
adamantane	19.145	19.551	16.281	14.430	13.896
ammonia	10.274	10.238	9.764	9.144	8.536
anthracene	25.692	29.750	23.116	23.977	24.713
benzene	13.081	14.522	11.896	11.812	11.869
CO_2	5.651	6.746	5.852	6.270	6.377
cyanamide	22.265	21.545	21.411	19.870	19.006
cytosine	38.782	40.945	36.734	36.829	37.030
ethylcarbamate	21.955	22.725	20.340	19.601	19.435
formamide	19.794	20.513	19.051	18.815	18.585
hexamine	21.722	23.845	20.094	19.369	19.319
imidazole	22.426	23.441	21.387	21.184	20.755
naphthalene	19.236	21.953	17.316	17.693	18.109
α oxalic acid	22.986	25.657	21.882	23.020	23.923
β oxalic acid	23.561	25.592	22.544	23.024	23.328
pyrazine	15.028	16.678	14.397	14.554	14.483
pyrazole	19.752	20.908	18.658	18.549	18.237
succinic acid	32.934	34.351	30.912	30.083	29.932
triazine	13.728	15.336	13.392	13.610	13.943
trioxane	15.310	17.008	14.173	14.075	14.209
uracil	33.074	35.205	31.518	31.781	32.117
urea	26.387	26.972	24.976	24.394	24.539
MAE	0.936	1.972	1.050	1.013	1.002
ME	0.509	1.947	-0.787	-0.979	-0.946
ice Ih	16.908	16.551	16.594	15.896	14.413
ice II	16.222	16.192	15.709	15.138	13.971
ice III	16.322	16.101	15.928	15.249	13.861
ice IV	15.725	15.764	15.210	14.644	13.538
ice VI	15.612	15.677	15.063	14.499	13.424
ice VII	14.248	14.632	13.630	13.245	12.521
ice VIII	14.533	14.903	13.907	13.507	12.787
ice IX	16.476	16.270	16.042	15.375	14.003
ice XI	16.977	16.615	16.665	15.960	14.471
ice XIII	16.075	16.017	15.565	14.940	13.735
ice XIV	15.910	15.890	15.379	14.759	13.591
ice XV	15.612	15.678	15.063	14.498	13.423
ice XVII	16.605	16.267	16.292	15.620	14.178
MAE	2.181	2.130	1.706	1.112	0.253
ME	2.181	2.130	1.706	1.112	-0.074

Table S7: X23 and ICE13 lattice energies, in kcal/mol, computed with selected methods using B86bPBE-XDM/light geometries.

Name	B86bPBE-XDM tight	B86bPBE25-XDM light	B86bPBE50-XDM light
1,4-cyclohexanedione	21.447	20.494	20.979
acetic acid	17.593	17.031	17.067
adamantane	17.734	15.173	14.512
ammonia	9.906	9.058	8.413
anthracene	25.182	25.380	25.911
benzene	12.706	12.298	12.309
CO_2	5.733	6.216	6.382
cyanamide	21.436	19.941	19.230
cytosine	37.770	37.436	37.649
ethylcarbamate	20.780	19.761	19.621
formamide	19.291	18.846	18.632
hexamine	22.589	20.232	19.801
imidazole	21.836	21.392	21.012
naphthalene	18.776	18.667	18.965
α oxalic acid	22.446	23.328	24.246
β oxalic acid	23.070	23.314	23.657
pyrazine	15.112	15.017	14.946
pyrazole	19.149	18.785	18.508
succinic acid	31.870	30.516	30.397
triazine	13.966	14.015	14.394
trioxane	15.243	14.516	14.501
uracil	32.202	32.221	32.600
urea	25.393	24.428	24.541
MAE	0.710	0.657	0.695
ME	-0.015	-0.588	-0.579
ice Ih	16.352	15.561	14.160
ice II	15.763	14.958	13.763
ice III	15.806	14.990	13.638
ice IV	15.362	14.506	13.349
ice VI	15.252	14.380	13.232
ice VII	14.142	13.246	12.373
ice VIII	14.429	13.510	12.641
ice IX	15.932	15.124	13.787
ice XI	16.422	15.625	14.220
ice XIII	15.629	14.771	13.536
ice XIV	15.499	14.615	13.397
ice XV	15.252	14.380	13.232
ice XVII	16.072	15.296	13.925
MAE	1.772	0.930	0.315
ME	1.772	0.930	-0.278

Table S8: X23 and ICE13 lattice energies, in kcal/mol, computed with selected composite methods involving basis-set correction using GGA/light (PBE-MBD, PBE-XDM, or B86bPBE-XDM) geometries.

Name	PBE0-MBD	PBE0-XDM	PBE50-XDM	B86bPBE25-XDM	B86bPBE50-XDM
1,4-cyclohexanedione	22.949	20.607	21.127	21.612	22.096
acetic acid	18.449	17.130	17.114	17.339	17.375
adamantane	19.625	16.003	15.469	17.294	16.633
ammonia	9.535	8.878	8.270	8.961	8.316
anthracene	27.904	24.382	25.118	26.296	26.827
benzene	13.762	12.154	12.211	12.931	12.941
CO_2	5.883	5.894	6.000	5.854	6.021
cyanamide	21.247	20.256	19.392	20.461	19.750
cytosine	39.389	36.776	36.978	37.806	38.019
ethylcarbamate	22.070	19.963	19.797	20.386	20.246
formamide	19.626	18.619	18.389	18.851	18.636
hexamine	22.626	20.053	20.003	21.914	21.482
imidazole	22.316	20.943	20.514	21.433	21.053
naphthalene	20.698	18.115	18.531	19.491	19.788
α oxalic acid	23.973	22.443	23.346	22.981	23.898
β oxalic acid	23.909	22.445	22.749	22.966	23.310
pyrazine	15.345	14.329	14.259	15.043	14.972
pyrazole	19.727	18.299	17.987	18.805	18.527
succinic acid	33.029	30.288	30.137	31.182	31.063
triazine	14.343	13.652	13.984	14.259	14.638
trioxane	15.882	14.157	14.291	14.951	14.936
uracil	33.787	31.713	32.049	32.426	32.805
urea	26.504	24.821	24.965	25.138	25.250
MAE	1.068	0.959	0.871	0.480	0.530
ME	0.913	-0.855	-0.822	-0.139	-0.130
ice Ih	14.963	14.592	13.109	14.383	12.983
ice II	14.678	14.053	12.886	14.034	12.838
ice III	14.526	14.037	12.649	13.912	12.560
ice IV	14.254	13.607	12.501	13.651	12.495
ice VI	14.189	13.507	12.433	13.570	12.422
ice VII	13.197	12.413	11.689	12.681	11.808
ice VIII	13.499	12.707	11.987	12.982	12.113
ice IX	14.716	14.177	12.805	14.065	12.727
ice XI	15.025	14.656	13.167	14.448	13.042
ice XIII	14.509	13.878	12.673	13.868	12.632
ice XIV	14.379	13.721	12.552	13.743	12.525
ice XV	14.189	13.506	12.431	13.571	12.422
ice XVII	14.701	14.333	12.891	14.136	12.765
MAE	0.612	0.305	1.161	0.190	1.195
ME	0.612	0.024	-1.161	0.013	-1.195
